# The causal effect of obesity on concomitant exotropia: A lifecourse Mendelian randomization study

**DOI:** 10.1097/MD.0000000000037348

**Published:** 2024-03-01

**Authors:** Changyang Liu, Yaxin Zhao, Jiasu Liu, Qi Zhao

**Affiliations:** a Department of Ophthalmology, the Second Hospital of Dalian Medical University, Dalian, China.

**Keywords:** concomitant exotropia, Mendelian randomization, obesity

## Abstract

Obesity is now a significant global public health issue. Limited understanding exists regarding the association between obesity and concomitant exotropia. Our objective was to identify the causal relationship between lifecourse obesity, including birth weight, childhood body mass index (BMI), and adult BMI, and the risk of concomitant exotropia. We used a two-sample Mendelian randomization (MR) strategy to examine the causal relationship with inverse-variance weighted method as the primary MR analysis. We carried out sensitivity analyses to evaluate the accuracy and robustness of our findings. Also, we performed reverse-direction MR analysis to eliminate the possibility of reverse causality. Childhood BMI, as opposed to birth weight or adult BMI, had a significant impact on the risk of concomitant exotropia (odds ratio = 1.40, 95% confidence interval (CI): 1.08–1.81, *P* = .01). This significance persisted even after accounting for birth weight and adult BMI using multivariable MR analysis (odds ratio = 1.35, 95% CI: 1.04–1.75, *P = *.02). There was no significant heterogeneity or pleiotropy observed in sensitivity analyses (*P* > .05). Multivariable MR analysis further confirmed the absence of pleiotropic effects of some risk factors including prematurity, maternal smoking around birth and refractive error. Reverse causality did not affect the causal relationship (beta = −0.0244, 95% CI: −0.0545 to 0.0056, *P* = .11). Genetic predisposition to higher childhood BMI was found to be causally linked to an increased risk of concomitant exotropia.

## 1. Introduction

Concomitant exotropia is a form of divergent strabismus whose deviating angle remains unaffected by gaze direction. This condition is often accompanied by closure of one eye, asthenopia, and diplopia.^[1–3]^ Moreover, it has been found to increase the risk of mental disorders^[4,5]^ and impair binocular vision. The pooled prevalence (95% confidence interval (CI)) of exotropia was estimated to be 1.23% (1.00–1.46).^[6]^ Determining the causal relationships between modifiable risk factors and concomitant exotropia holds essential practical implications in the investigation of the disease’s etiology, as well as in its prevention and management within the realm of public health. Prior studies have identified various potential risk factors for concomitant exotropia, such as being female, having a family history of strabismus, maternal smoking during pregnancy, prematurity, experiencing hypoxia at birth, low birth weight, and refractive error.^[7–11]^

Obesity is a widespread problem that results in significant illness and death. In the last forty years, there has been a significant rise in the prevalence of childhood obesity, emerging as a critical public health concern worldwide.^[12]^ In our clinical observations, we have noted a high occurrence of obesity among patients with concomitant exotropia, but there is limited knowledge regarding the association between lifecourse obesity (birth weight, childhood body mass index (BMI), and adult BMI) and concomitant exotropia. A systematic review has demonstrated that low birth weight was a notable risk factor for strabismus.^[11]^ Nevertheless, it is important to consider potential confounding factors, reverse causality, misclassification, and other biases when interpreting this finding.

Mendelian randomization (MR) has emerged as a valuable approach to address these concerns. MR is an approach employed in the domain of epidemiological etiology to establish causal relationships. By incorporating instrumental variables as genetic predictors, this approach mitigates the impact of residual confounding factors since genetic variants are randomly assigned during conception.^[13]^ Additionally, MR designs offer the advantage of minimizing the potential for reverse causality, as genetic variants remain unaltered by the onset and progression of diseases. As an extension of univariable MR, multivariable MR enables the simultaneous assessment of genetically predicted impacts of multiple risk factors on a given outcome.^[14]^ In this study, we used the two-sample MR framework incorporating sensitivity analyses to explore the causal association between lifecourse obesity and concomitant exotropia.

## 2. Methods

### 2.1. Study design

This study was conducted based on the 3 fundamental assumptions of MR, namely relevance, independence, and exclusion assumptions.^[15]^ Specifically, the chosen instrumental variables exhibited a strong association with the exposure, but not any confounding variables or the outcome. Univariable MR analysis was employed using the published genome-wide association study (GWAS) summary statistics to examine the causal relationship between lifecourse obesity and concomitant exotropia. Reverse-direction MR analysis was utilized to account for the possibility of reverse causality. Additionally, multivariable MR analysis was performed to assess the “direct” effect of lifecourse obesity on concomitant exotropia and to investigate potential pleiotropic effects. Sensitivity analysis was carried out to verify the robustness of the findings. As far as we know, there was no sample overlap between the exposure and outcome GWASs, because they were obtained from distinct study populations, as mentioned below.

### 2.2. Data sources

The instrumental variables related to birth weight were acquired from a GWAS meta-analysis using data from the UK Biobank and the Early Growth Genetics consortium.^[16]^ The dataset included approximately 92.81% of European individuals. The instrumental variables related to childhood BMI were obtained from a GWAS meta-analysis contributed by the Early Growth Genetics consortium, which combined data from 26 studies.^[17]^ The instrumental variables related to adult BMI were derived from a GWAS meta-analysis that integrated information from the UK Biobank and the Genetic Investigation of Anthropometric Traits consortium.^[18]^ The GWAS summary statistics for concomitant exotropia (phenocode: H7_DIVERGSTRAB) were acquired from the FinnGen consortium (r9.finngen.fi).^[19]^ Detailed characteristics of these GAWS datasets were shown in Table 1.

**Table 1:** Characteristics of genome-wide association study summary statistics.

Trait	Year	Ethnicity	Sample size	Number of SNPs	Source
Birth weight	2019	Mainly European	321,223	14,857,286	EGG consortium
Childhood BMI	2020	European	39,620	8226,429	EGG consortium
Adult BMI	2018	European	694,649	2529,254	GIANT consortium
Concomitant exotropia	2023	European	363,526	20,169,990	FinnGen consortium
Preterm birth	2019	European	64,923	7545,602	EGG consortium
Maternal smoking around birth	2018	European	397,732	9851,867	IEU OpenGWAS project
Refractive error	2018	European	361,194	13,770,980	UK Biobank

BMI = body mass index, EGG = Early Growth Genetics, GIANT = Genetic Investigation of Anthropometric Traits, SNPs = single-nucleotide polymorphisms.

### 2.3. Selection of instrumental variables

The genome-wide significant (*P* < 5 × 10^−8^) single-nucleotide polymorphisms (SNPs) were filtered in the GWAS for lifecourse obesity. To prevent linkage disequilibrium, a strict clump window (*r*^2^ < 0.001 within 10,000 kb) was applied. The Steiger method was employed to ensure the included SNPs explained more variance in exposure compared to outcome, thereby confirming the robustness of the causal relationship.^[20]^ In the reverse-direction MR analysis, only 2 SNPs were found to be related to concomitant exotropia based on the threshold requirement of *P* < 5 × 10^−8^. Since a minimum of 10 SNPs are required for a MR study,^[21]^ we selected instrumental variables with *P* < 5 × 10^−6^ to obtain 16 SNPs for MR analysis. We utilized the phenoscanner website (http://www.phenoscanner.medschl.cam.ac.uk/)^[22]^ to exclude the SNPs associated with potential confounding variables, using a significance threshold of *P* < 1 × 10^−5^. The strength of each genetic instrument was assessed using the F-statistic according to the approach outlined by Pierce et al.^[23]^ SNPs with an F-statistic > 10 were considered as strong instruments.^[23,24]^ To estimate the statistical power, a web-based tool (https://shiny.cnsgenomics.com/mRnd/) was used.^[25]^

### 2.4. Statistical analysis

The statistical analysis was conducted using R software version 4.3.1 (https://www.r-project.org/) with TwoSampleMR (version 0.5.7). The principal analyses were performed utilizing the inverse-variance weighted (IVW) method with a random-effect model, which provided a reliable and accurate causal assessment.^[26]^ Additionally, MR-Egger regression (MER), weighted median, and weighted mode were employed to estimate causal effects. In comparison to IVW method, the 3 methods exhibit diminished statistical power,^[27]^ thus rendering them supplementary approaches in this study. To evaluate heterogeneity, Cochrane’s Q-statistic was utilized, with significant heterogeneity being identified if *P* < .05.^[28]^ To evaluate the stability of the MR results, the leave-one-out sensitivity test was conducted by excluding instrumental variables one by one.^[29]^ Furthermore, the effects of outlying instrumental variables were assessed by the MR pleiotropy residual sum and outlier (MR-PRESSO) distortion test.^[30]^ Based on the MR-PRESSO global test and the MER analysis intercept, directional pleiotropy was examined.^[31]^ Causal estimates were presented as odds ratios (ORs) and 95% CIs. Statistical significance was determined by considering a significance level of 0.017, which was adjusted using the Bonferroni correction (*P* = .05/3).

Obesity at 3 different stages of life was included in the multivariable MR analysis to examine the “direct” effect on concomitant exotropia. Furthermore, several GWAS summary statistics (Table 1) for other potential risk factors of concomitant exotropia, including preterm birth,^[32]^ maternal smoking around birth, and refractive error, were also included to adjust for the potential pleiotropic effects.^[14,33]^ All SNPs significantly associated with any exposure were pooled in the multivariable MR analysis, and then clumped based on linkage disequilibrium (*r*^2^ < 0.001 within 10,000 kb).

### 2.5. Ethical approval

Ethical permit was not needed for this study because we only used openly available GWAS summary data and did not have access to individual-level information. The related original studies have obtained ethical permits and written informed consent.

## 3. Results

### 3.1. Instrumental variables

All the genetic instruments for birth weight (152 SNPs) and childhood BMI (17 SNPs) met the independence assumption, the selected SNPs not associated with confounding factors. But 5 SNPs (rs12150665, rs2066295, rs215669, rs3796432, and rs8097672) related to adult BMI were removed from further analysis due to their association with confounding factors, maternal smoking around birth. Consequently, 152, 17, and 483 SNPs were included in the final analyses, which explained 2.80%, 2.66%, and 4.68% of variability in these traits, respectively. All F-statistics exceeded 10, indicating the absence of significant weak instrument bias. Details of the included SNPs can be seen in Tables S1–S3, Supplemental Digital Content, http://links.lww.com/MD/L832, http://links.lww.com/MD/L833, http://links.lww.com/MD/L834. The power was low for the analyses of birth weight and adult BMI, while it was adequate (0.88) for the analysis of childhood BMI.

### 3.2. Univariable MR

**Figure 1.**
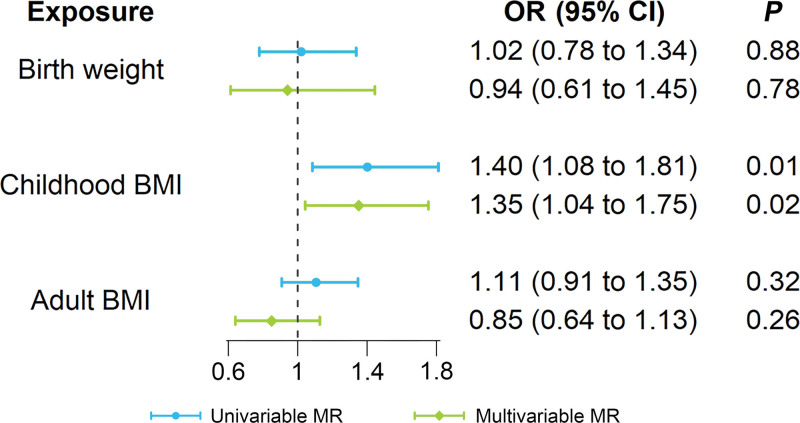
Forest plot of univariable and multivariable MR for lifecourse obesity effects on concomitant exotropia. BMI = body mass index, CI = confidence interval, MR = Mendelian randomization, OR = odds ratio.

The ORs of concomitant exotropia were 1.02 (95% CI: 0.78–1.34, *P* = .88) and 1.11 (95% CI: 0.91–1.35, *P* = .32) for 1-standard deviation increase in birth weight and adult BMI, respectively (Fig. 1). There was no heterogeneity detected in the analysis of the 2 exposures (*P* for Cochrane’s Q > 0.05). Furthermore, there was no evidence of pleiotropy found in the MER analysis (*P* for pleiotropy > 0.05) or in the MR-PRESSO global test (*P* for global test > 0.05).

**Figure 2.**
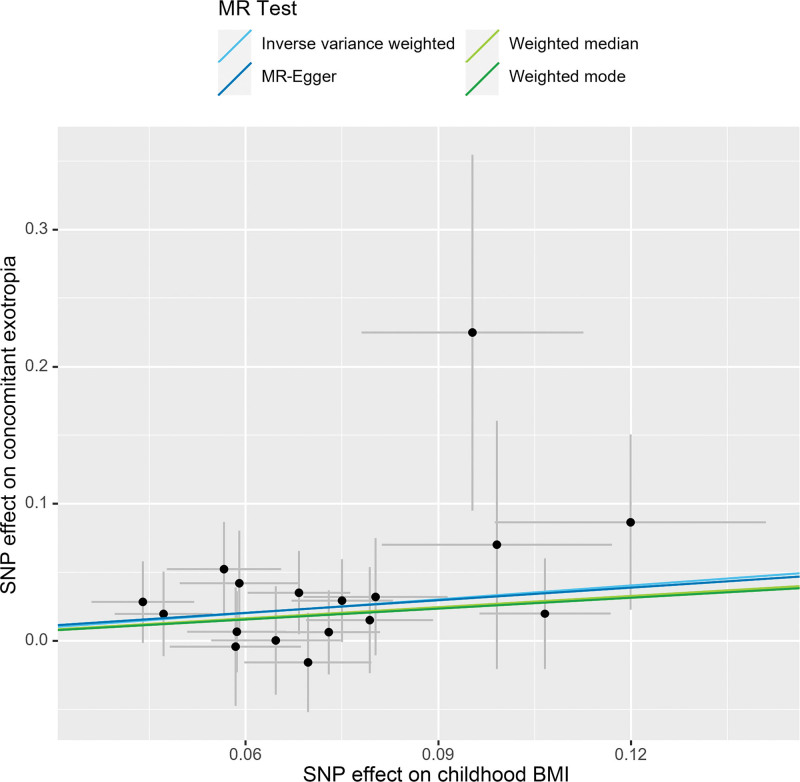
Scatter plot to visualize the causal relationship between childhood BMI and concomitant exotropia. BMI = body mass index, MR = Mendelian randomization, SNP = single-nucleotide polymorphism.

**Figure 3.**
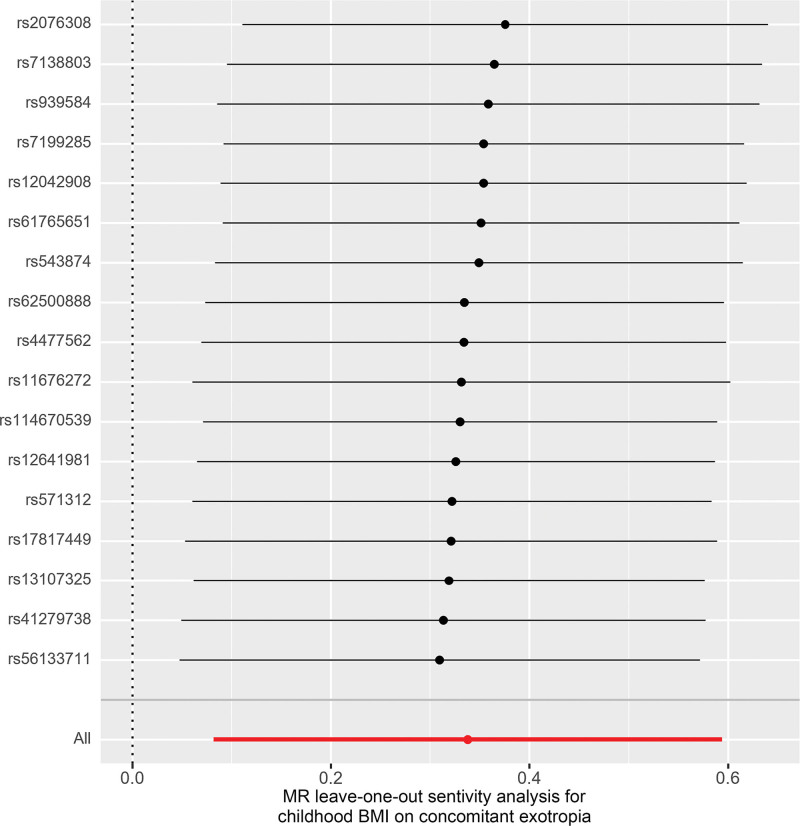
Leave-one-out plot of childhood BMI on the risk of concomitant exotropia. BMI = body mass index, MR = Mendelian randomization.

Higher childhood BMI, as predicted by genetic factors, was associated with an elevated risk of concomitant exotropia (Fig. 2). In the main analysis, the OR of concomitant exotropia was 1.40 (95% CI: 1.08–1.81, *P* = .01) with each standard deviation increase in childhood BMI (Fig. 1). The association between different methods (MER, weighted median, and weighted mode) remained directionally consistent, although *P* values for them were above 0.05. The results were robust according to sensitivity analyses. Heterogeneity test indicated no significant heterogeneity (Cochrane’s Q = 7.1, *P* = .97). MER analysis indicated no evidence of directional pleiotropy (intercept 0.0022, *P* = .95). MR-PRESSO analysis detected no outlier, and the *P* value for global test was greater than 0.05, suggesting the absence of horizontal pleiotropy. The exclusion of any of the 17 SNPs did not alter the association direction (Fig. 3).

### 3.3. Reverse-direction MR

For reverse-direction analysis, 2 SNPs (rs35123409 and rs79815018) were excluded from the genetic instruments for concomitant exotropia, due to their association with height or weight. The F-statistics for the remaining 14 SNPs were all above 10, indicating the lack of strong evidence for weak instrument bias. Details of the included SNPs can be seen in Table S4, Supplemental Digital Content, http://links.lww.com/MD/L835. Our analysis revealed limited evidence suggesting the reverse causality of genetic liability for concomitant exotropia on childhood BMI (beta = −0.0244 per 1-standard deviation change in concomitant exotropia liability, 95% CI: −0.0545 to 0.0056, *P* = .11). These findings suggested that the effect of childhood BMI on concomitant exotropia risk could not be explained by reverse causality.

### 3.4. Multivariable MR

Multivariable MR analysis with IVW method provided evidence suggesting the risk association for childhood BMI on concomitant exotropia (OR = 1.35, 95% CI: 1.04–1.75, *P* = .02) remained significant, albeit weakened in comparison with univariable MR analysis, after controlling for birth weight and adult BMI (Fig. 1). Additionally, multivariable estimates provided limited evidence for a “direct” effect of birth weight or adult BMI on concomitant exotropia risk when considering childhood BMI in the multivariable framework (Fig. 1). There was no significant heterogeneity observed in the evaluation of multivariable IVW method (*P* = .11). In multivariable MR analysis adjusting for other potential risk factors, including preterm birth, maternal smoking around birth and refractive error, the causal association between childhood BMI and concomitant exotropia was unchanged (OR = 1.40, 95% CI: 1.08–1.81, *P* = .01), suggesting the absence of pleiotropic effect on the causal association.

## 4. Discussion

This is the first MR study to conduct a comprehensive evaluation of the causal relationship between lifecourse obesity and the risk of concomitant exotropia. We identified 152, 17, and 483 SNPs using 3 GWAS datasets (birth weight, childhood BMI, and adult BMI). Employing 4 different models, we successfully demonstrated a robust causal relationship between childhood BMI and the risk of concomitant exotropia. The sensitivity analyses performed in our study reinforced the robustness and accuracy of our causal conclusion. Moreover, the reverse-direction MR analysis effectively eliminated the possibility of reverse causality influencing our findings. Multivariable MR analysis further verified the findings of univariable MR analysis and the absence of pleiotropic effects. These results provided the evidence that the genetic predisposition for childhood BMI, rather than birth weight or adult BMI, exhibited a causal association with concomitant exotropia. Consequently, early detection for concomitant exotropia may be warranted for children with elevated BMI.

The effect of genetically predicted childhood BMI on the risk of concomitant exotropia could have various explanations. From a functional standpoint, obesity was shown to impact the contractile function of skeletal muscles,^[34–36]^ which could potentially disrupt the balance of extraocular muscle forces and ultimately contribute to the development of concomitant exotropia. On a structural level, a light microscopy study demonstrated the presence of medial rectus atrophy in individuals with concomitant exotropia.^[37]^ Furthermore, Hao et al^[38]^ found patients with concomitant exotropia exhibited statistically smaller medial rectus sizes by using high-resolution MRI. It is hypothesized that obesity-induced muscle atrophy^[39–43]^ may be a contributing factor to the development of concomitant exotropia. At the molecular level, matrix metalloproteinases (MMPs) showed a positive correlation with the severity of intermittent exotropia, while tissue inhibitors of metalloproteinase (TIMPs) showed a negative correlation.^[44]^ Obese individuals exhibited significantly higher concentrations of MMPs in their saliva compared to individuals with normal body weight.^[45]^ Furthermore, there was a correlation between plasma concentrations of MMPs and TIMPs with anthropometric parameters in obesity.^[46]^ Consequently, it is plausible that obesity may induce concomitant exotropia through its impact on MMPs and TIMPs. Irrespective of the specific mechanisms involved, our findings indicated the presence of a critical period during childhood in which the influence of obesity on concomitant exotropia could be mitigated.

We found that birth weight was not the causal factor for the effect of childhood body size on the risk of concomitant exotropia in our model. Previous observational studies have documented an inconclusive correlation between birth weight and strabismus. A large multidisciplinary cohort study encompassing 39,227 children revealed that low birth weight was an independent risk factor for both esotropia and exotropia.^[10]^ Conversely, a cross-sectional study involving 563 children between the ages of 4 and 10 suggested that birth weight percentile exerted a lesser independent impact on strabismus,^[47]^ aligning with our findings. The previous contradictory results may be attributed to selection bias stemming from the composition of strabismus subtypes, limited sample size, and suboptimal study designs (prone to the confounding factors from cross-sectional or cohort designs). Our MR analysis presents a significant advantage in that the causal inference drawn through genetic instruments is less susceptible to confounding factors and reverse causality. In addition, our results did not support that obesity later in life played a role in influencing concomitant exotropia. Considering the early onset of concomitant exotropia, it was expected that the effect of adult body size on the risk of this condition was not significant.

The primary strengths of our study lie in the utilization of a MR design and the inclusion of a substantial number of patients with concomitant exotropia. Furthermore, our findings exhibited consistency across several sensitivity analyses. The Steiger method provided support for the influence of selected SNPs on our exposure prior to our outcome. Meanwhile, the reverse-direction MR analysis did not support reverse causality. Furthermore, we investigated the effect of obesity on concomitant exotropia from a longitudinal standpoint. The finding that childhood BMI, as predicted by genetic factors, exhibited a causal relationship with concomitant exotropia, while birth weight and adult BMI did not, implies the necessity of increased focus on children with elevated BMI to mitigate the development of concomitant exotropia.

There are some limitations to our work. Firstly, it is important to note that the associations observed in this study have not been validated in other cohorts due to the current absence of GWAS data. Consequently, the findings should be cautiously interpreted. When qualified relevant GWAS data become accessible, further confirmation of the findings is needed. Secondly, our findings may be biased by potential horizontal pleiotropy, the impact of which cannot be completely ruled out, as horizontal pleiotropy may be widespread across the genome. Nevertheless, we conducted multiple sensitivity analyses (including MR-Egger intercept, MR-PRESSO analysis, and multivariable MR analysis) to detect and correct for horizontal pleiotropy, and we did not find any evidence of its presence. Thirdly, the GWASs used in this study primarily consist of European individuals. Thus, it is difficult to generalize our findings to other races. Fourthly, birth weight can be further dissected into the effects stemming from maternal and fetal genetics.^[16]^ However, our study specifically focused on investigating the fetal genetic aspect. Consequently, it is necessary for future research endeavors to thoroughly examine the causal relationship between birth weight and strabismus. Finally, the influence of obesity on other types of strabismus is not yet fully understood. Hence, additional validation studies are required to investigate the correlation between obesity and other forms of strabismus.

To conclude, we presented MR evidence that supported a causal relationship between childhood BMI and the risk of concomitant exotropia. Our findings suggested children with elevated BMI levels should receive targeted clinical intervention to mitigate the risk of developing concomitant exotropia. Additional studies are necessary to investigate the fundamental biological mechanisms that contribute to this association.

## Acknowledgments

The authors want to acknowledge the participants and investigators of the EGG consortium, GIANT consortium, FinnGen consortium, UK Biobank, and IEU OpenGWAS project.

## Author contributions

**Conceptualization:** Changyang Liu, Yaxin Zhao, Qi Zhao.

**Data curation:** Changyang Liu.

**Formal analysis:** Changyang Liu, Yaxin Zhao, Jiasu Liu.

**Investigation:** Changyang Liu.

**Methodology:** Changyang Liu.

**Project administration:** Qi Zhao.

**Resources:** Changyang Liu, Qi Zhao.

**Software:** Changyang Liu.

**Supervision:** Qi Zhao.

**Validation:** Changyang Liu, Yaxin Zhao, Jiasu Liu, Qi Zhao.

**Visualization:** Changyang Liu.

**Writing – original draft:** Changyang Liu, Qi Zhao.

**Writing – review & editing:** Changyang Liu, Yaxin Zhao, Jiasu Liu, Qi Zhao.

## Supplementary Material








